# Potential of Time Domain Nuclear Magnetic Resonance as a Noninvasive Method for Detection and Quantification of Protein Glycation in Biopharmaceuticals

**DOI:** 10.1021/acs.analchem.5c07921

**Published:** 2026-03-02

**Authors:** Hani Alam, Ozlem Gezici Koc, Cem Yamali, Mecit Halil Oztop

**Affiliations:** a Department of Biotechnology, Graduate School of Natural and Applied Sciences, 52984 Middle East Technical University, Cankaya, Ankara 06800, Türkiye; b Department of Food Engineering, Faculty of Engineering, 52984 Middle East Technical University, Çankaya, Ankara 06800, Türkiye; c Scientific and Technological Research Council of Türkiye (TÜBİTAK), Ankara 06100, Türkiye; d Department of Basic Pharmaceutical Sciences, Faculty of Pharmacy, 37506 Çukurova University, Sarıçam, Adana 01330, Türkiye

## Abstract

Biopharmaceuticals are an essential and growing part of modern medicine. Given their complex structure, they are prone to chemical and physical instabilities, including glycation, a nonenzymatic chemical reaction between the free amino groups of proteins and reducing saccharides. Glycation reduces the drug’s efficacy and can produce harmful compounds in the body. Current detection methods, such as liquid chromatography–mass spectrometry, affinity chromatography, and chemical reaction methods, including the *O*-phthalaldehyde (OPA) method, are invasive, labor-intensive, and costly. This study presents time domain nuclear magnetic resonance (TD-NMR) as a noninvasive, low-cost, and user-friendly method for the detection and quantification of glycation in biopharmaceuticals. Bovine serum albumin and glucose were employed as a model system and were placed under various temperatures and durations to induce glycation. Several TD-NMR techniques, including one-dimensional longitudinal (*T*_1_) and transverse (*T*_2_) relaxation times, as well as 2-D *T*_1_ *T*_2_ maps, were applied to the glycated samples, providing a better understanding of the hydration behavior and water mobility during glycation. Comparing these results to the OPA method had shown that both *T*_1_ and *T*_2_ values change proportionally with glycation, indicating alterations in molecular mobility and hydration. *T*_1_/*T*_2_ map values were highly correlated with the OPA method (*p* < 0.05, RMSE ≤ 0.12, MAE ≤ 0.10), supporting the applicability of TD-NMR as a quantitative glycation measurement tool. Additional analyses, such as protein quantification, secondary structure evaluation, and assessment of early and late glycation products, further confirmed the relevance of TD-NMR in monitoring structural and chemical changes during glycation.

## Graphical abstract

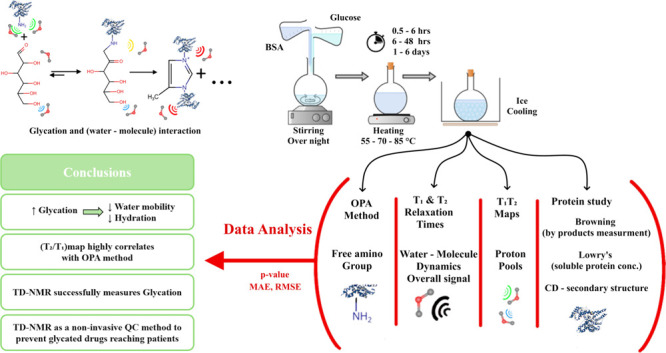

## Introduction

Biopharmaceuticals have witnessed substantial growth in recent decades, primarily due to significant advancements in recombinant DNA technology. They are prominent in the treatment of a wide range of conditions such as cancer, asthma, central nervous system ailments, infections, and cardiovascular illnesses.^1,2^ Of these, hundreds have been approved and commercialized, with some being among the top-selling pharmaceuticals. Biopharmaceuticals constitute over one-third of drugs approved by the FDA, with projections indicating that their market value will be around $400 billion by 2025.^3–5^ Nonetheless, their commercialization and broad adoption face a vital issue in their physical and chemical instabilities. Aggregation and chemical modifications can alter their protein structure, leading to changes in efficacy, immunogenicity, and toxicity.^6–8^ These illustrate the importance of stability studies, which investigate drugs’ quality under various environmental and storage conditions, and validate the quality, efficacy, and safety of biopharmaceuticals.^9^

One of the factors contributing to these instabilities is the glycation reaction. Glycation is a nonenzymatic chemical reaction between a protein’s primary amine molecule and the aldehyde unit of a reduced saccharide (Figure 1). This process can affect the functionality and reliability of biopharmaceuticals by changing their protein structure and pharmacokinetics, thereby lowering the efficacy of protein-based drugs.^10–13^ Consequently, it reduces the expected benefits of these drugs or forms inhomogeneous drug effects between batches. As demonstrated in Figure 1, glycation reaction starts with an interaction between a reducing saccharide and a protein molecule, such as protein-based biopharmaceuticals, forming a complex compound known as a Schiff base. Following this initial reaction, the Schiff base undergoes additional transformations in the sequential order of dehydration, rearrangement, cyclization, oxidation, and another round of dehydration. These processes progress into advanced glycation end-products (AGEs), which are a group of highly stable compounds.^14,15^ AGEs are harmful, and their accumulation may lead to several health conditions such as diabetes, chronic kidney failure, aging, and Alzheimer’s disease.^16–20^ The damaging effects of AGEs have led to numerous studies to find preventive treatments for AGE formation or treatments to remove these harmful compounds from the body.^21,22^ However, these treatments have not yet been standardized.^23^

**Figure 1:**
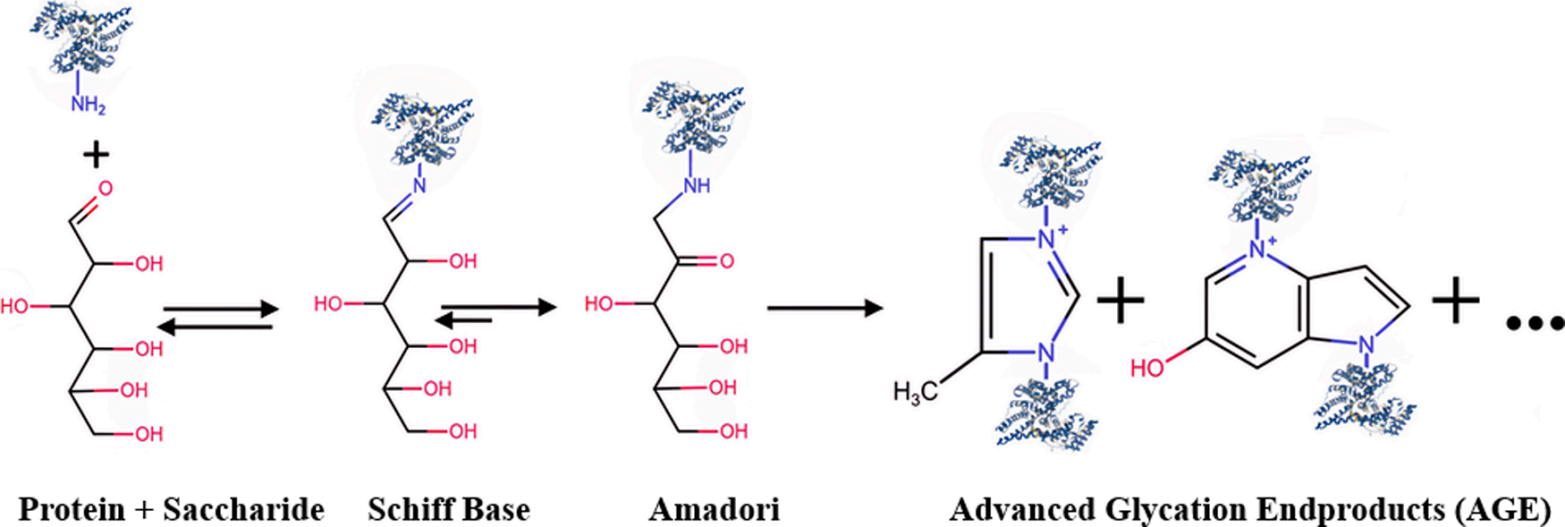
Glycation reaction and its following reactions and products.

Glycation may occur at various stages of drug production, including the fermentation stage, as glucose is used for cell growth, or during the downstream stage due to the presence of sugars. The reactivity of these reactions depends on the therapeutic protein involved. The rate and extent of glycation can be influenced by factors such as temperature, pH, duration, and ionic strength, which should be maintained at physiological levels.^12,24–26^ To reduce glycation risk, nonreducing disaccharides such as sucrose and trehalose are widely used as excipients in biopharmaceuticals due to their stabilizing properties and their ability to preserve antibodies and proteins.^1,27,28^ However, glycation may still occur throughout storage, even with nonreducing saccharides. This can develop under high temperatures or acidic environments, which hydrolyze nonreducing saccharides to form the reducing saccharide glucose.^12,29,30^

Quality assurance is fundamental to ensure the efficacy and integrity of drugs and prevent glycated drugs and biopharmaceuticals containing advanced glycation end-product (AGEs) from reaching patients. Quality assurance includes detection methods implemented during and after production, as well as during storage and transportation.^13,31,32^ Various techniques are available for identifying glycation in biopharmaceuticals. These include liquid chromatography–mass spectrometry (LC–MS), which measures overall glycation levels and identifies specific glycation sites through peptide mapping.^32^ Boronate affinity chromatography is another method used for glycation measurements. The specific interactions between the boronate groups of the stationary phase and *cis*-diol groups in glycated proteins determine their selective capture and separation.^13^ Moreover, fluorescence analysis can be used to measure glycation.^33^ Furthermore, chemical calibration procedures can be employed, such as the *O*-phthalaldehyde (OPA) method, which is one of the most commonly used glycation measurement methods. It quantifies glycation by involving the reaction of free amino groups in the protein with the resulting OPA, resulting in a derivative that can be measured using a spectrophotometer. The reduction in free amino groups is directly correlated to the extent of glycation.^33,34^

Conventional analytical techniques for glycation measurements, such as mass spectrometry and fluorescence assays, are fundamentally invasive. They require opening the pharmaceutical containers and often involve sample consumption, solvent addition, or the usage of external probes,^35–37^ any of which would turn the tested material unsuitable for patients. Consequently, only a limited number of samples can be analyzed per batch, meanwhile, extrapolating the results to the remaining units. Meanwhile, TD-NMR is a noninvasive technique that enables the analysis of biopharmaceutical formulations without consuming any part of the sample, adding chemicals, or opening sealed containers. Entire packaged units, such as sealed vials, can be placed directly into the instrument, measured, and subsequently returned to storage or clinical use if no quality issue is detected. This nondestructive nature allows screening of all units within a batch, making TD-NMR particularly attractive for high-throughput quality control applications.^38,39^

As a result, only a small subset of samples from each batch can be tested, limiting assessments of inter- and intrabatch consistency. In contrast, a noninvasive method preserves the integrity of the drug, allowing it to be applied across all samples in each batch to measure interbatch and intrabatch differences without sacrificing any sample,^40^ therefore enhancing quality control and reducing related costs. Time domain NMR (TD-NMR) presents a promising alternative to these invasive methods since TD-NMR is a noninvasive, cost-effective, rapid (seconds to minutes per sample), mobile, and user-friendly method, with commercially available automation solutions enabling high-throughput analysis.^41,42^ Furthermore, the simplicity, compactness, and low cost of TD-NMR make it an excellent candidate for routine use, even in settings with limited resources. TD-NMR is based on measuring molecular mobility through magnetic relaxation times of longitudinal (*T*_1_) and transverse (*T*_2_) proton relaxations. This translates to measuring the molecular mobility of ^1^H nuclei, which are abundant in water, making TD-NMR an effective method for analyzing water mobility and water-drug molecular interactions in biopharmaceuticals. TD-NMR has been used in many scientific and industrial fields and particularly has increased in the pharmaceutical research field.^43,44^

The relationship between TD-NMR and glycation can be realized through three principal physicochemical outcomes of the glycation reaction. First, glycation involves a cascade of smaller reactions including condensation, rearrangement, polymerization, oxidation, dehydration, enolization, cyclization, and fragmentation.^45,46^ Collectively, they reduce the availability of free hydrogen-bonding sites on solutes such as proteins, saccharides, and glycation byproducts, and this in turn decreases the solute–water interactions and proton exchange, which directly influence TD-NMR relaxation behavior.^47–49^ Second, glycation is known to increase the hydrophobicity of protein surfaces,^50,51^ altering hydration layers and reducing water mobility at the protein interface. These changes are well established to affect longitudinal (*T*_1_) and transverse (*T*_2_) relaxation times.^52–54^ Finally, glycation promotes aggregation,^55,56^ which further restricts water mobility;^44,57^ reduced molecular mobility is a known determinant of TD-NMR signal changes. Based on these established physicochemical effects, the primary hypothesis of this study is that TD-NMR relaxation parameters through their sensitivity to hydration, water mobility, and water–polymer interactions are proportional to glycation progression, enabling TD-NMR to serve as an alternative, noninvasive measurement tool.

This study aims to address the limitations of the current glycation detection methods by offering TD-NMR as an alternative, novel, noninvasive, and low-cost method suitable for quality control of biopharmaceutical products. Glucose and bovine serum albumin were subjected to accelerated stress conditions to serve as a model system for glycated biopharmaceuticals. Then, their glycation was measured through several TD-NMR techniques, namely, *T*_1_ longitudinal relaxation, *T*_2_ transverse relaxation, and *T*_1_ *T*_2_ maps. The results were compared to each other and toward two conventional glycation detection techniques, Browning measurement and OPA method, thereby establishing the accuracy and reliability of TD-NMR for glycation measurements. Furthermore, protein quantification and protein’s secondary structure were studied to gain a more profound understanding of the relationship between TD-NMR and proteins during glycation. In addition, TD-NMR studies have provided a more profound understanding of the hydration behavior and water mobility of molecules during the glycation reaction.

## Experimental Section

### Materials

Sodium bicarbonate (NaHCO_3_), zinc sulfate heptahydrate (ZnSO_4_·7H_2_O), *o*-phthaldialdehyde (OPA), sodium dodecyl sulfate (SDS), sodium carbonate, sodium hydroxide, bovine serum albumin (BSA), sodium potassium tartrate tetrahydrate, sodium azide (NaN_3_), and β-mercaptoethanol (2-mercaptoethanol) were purchased from Sigma-Aldrich (Saint Louis, MO, USA). Glucose was acquired from Tito (Türkiye). Disodium tetraborate decahydrate (Na_2_B_4_O_7_·10 H_2_O), copper­(II) sulfate, potassium hexacyanoferrate­(II) trihydrate (K_4_[Fe­(CN)_6_]·3H2O), ethanol, and Folin–Ciocalteu’s phenol reagent were supplied by Merck KGaA (Darmstadt, Germany). Distilled water was obtained from a 0.2 μs/cm purity mpMinipure Dest system (mpMinipure Ultrapure Water Systems, Ankara, Türkiye).

### Sample Preparation and Glycation

Bovine serum albumin (20 mg/mL) was mixed with glucose (180 mg/mL) in distilled water, and sodium azide (0.02% w/v) was added to inhibit microbial growth. Similar BSA and glucose ratios have also been used in other studies.^58–60^ The prepared solutions were mixed and allowed to stand overnight. These were then divided into three groups, each of which was further divided into several samples representing different time points. The first group of samples had time points of (0, 0.5, 1, 2, 4, and 6 h). They were placed inside a water bath at 85 °C and closed tightly to avoid evaporation. The second group of samples was put inside a water bath at 70 °C, and their time points were chosen as follows: (0, 3, 6, 12, 24, 36, and 48 h). While samples of the third group were placed in a temperature-controlled cabinet at 55 °C for 6 days (0, 1, 2, 3, 4, 5, and 6 days). All samples were removed according to their time points and placed in water at room temperature for 30 min to stop further glycation. Three replicates were performed for each sample from each group. The samples were kept inside the refrigerator at 4 °C for further experiments, except at time point zero, which was not placed inside the water bath but directly in the refrigerator. For circular dichroism measurements, one sample (BSA) was prepared similarly to point zero but without glucose, and it is called native BSA.

### Quantification of Free Amino Groups

The quantification of available amino groups was measured using the OPA method with some modifications.^61,62^ The OPA reagent was prepared by using OPA (*O*-phthalaldehyde), ethanol, borax buffer, β-mercaptoethanol, and SDS (sodium dodecyl sulfate) solution. For the preparation, 40 mg of the OPA reagent was dissolved in 1 mL of 95% ethanol solution. After complete dissolution, 25 mL of 100 mM borax buffer (pH 9.5) was added to the solution. Reagent preparation was completed by the addition of 100 μL of β-mercaptoethanol and 2.5 mL of 20% SDS solution. Finally, the volume of the reagent was adjusted to 50 mL. After the preparation of the OPA reagent, one volume of 40× diluted samples was mixed with three volumes of the prepared OPA reagent and waited for 3 min. Absorbance was measured at 340 nm using a Microplate Reader (FlexA-200, Allsheng, Hangzhou, China).

### Browning Measurements

Glycation produces colorless intermediate products and brown products that indicate an advanced stage of the reaction. The UV–vis Microplate Reader (FlexA-200, Allsheng, Hangzhou, China) was used to measure intermediate products after 40× dilution at 294 nm, while Browning measurements were obtained without dilution at 420 nm.^63,64^

### Protein Solubility Measurement

Lowry’s method was adopted with some modifications to measure soluble protein quantities in samples.^65,66^ Lowry’s reagent was prepared by adding copper­(II) sulfate (1 mL 2% w/v) and sodium potassium tartrate (1 mL 2% w/v) to sodium carbonate (100 mL 2% w/v), which contained NaOH (0.4% w/v). Freshly prepared Lowry’s reagent (5 mL) was added to the diluted sample (1 mL, 10×), vortexed, and incubated for 10 min at room temperature, and after that, diluted Folin-Ciocalteu’s phenol reagent (0.5 mL, 2 N, 2×) was added to the mixture and mixed well by vortexing. The mixtures were incubated for 30 min in the dark at room temperature, and finally, they were measured at an absorbance of 680 nm in a UV–vis Microplate Reader (FlexA-200, Allsheng, Hangzhou, China).

### TD-NMR Measurements

The experimental setup was performed by using a benchtop NMR system (Pure Devices GmbH, Germany) operating at a ^1^H frequency of 24.15 MHz. The system was set up with a radio frequency coil measuring 10 mm, corresponding to the cylinder where the samples were placed inside 10 mm wide tubes. The device’s temperature was calibrated to 28 °C, and samples were adjusted to this temperature before measurements. All TD-NMR measurements were conducted at 28 °C to ensure consistent and reproducible experimental conditions, to minimize temperature dependent relaxation effects and to ensure that observed differences in relaxation behavior arise from sample composition rather than instrumental variability. Measuring at 28 °C, slightly above room temperature, enables stable operation of benchtop TD-NMR systems without affecting the integrity of sealed pharmaceuticals during any short measurement period.

### Longitudinal Relaxation Times (*T*_1_)

The *T*_1_ relaxation times of the glycated samples were determined using a saturation recovery (SR) sequence with the number of echoes as 2048, the echo time interval as 16.7 ms, the number of time points as 20, and the number of scans as 1. The obtained signals were analyzed using MATLAB software (ver. R2025a, The MathWorks Inc., 2025) and fitted to a monoexponential model to determine *T*_1_ relaxation times. In addition, the spectrum of *T*_1_ relaxation times was obtained by applying the inverse Laplace transform (T1-ILT).

### Transverse Relaxation Times (*T*_2_)

The *T*_2_ relaxation times of the glycated samples were examined by using the Carr–Purcell–Meiboom–Gill (CPMG) sequence. The number of echoes is 12,500, the echo time interval is 10 ms, the repetition time (TR) is 13000 ms, and the number of scans is 2. The NMR signals received for *T*_2_ relaxation times were evaluated using MATLAB software (ver. R2025a, The MathWorks Inc., 2025) and fitted into monoexponential models to acquire *T*_2_ relaxation times. In addition, the spectrum of *T*_2_ relaxation times was obtained by applying the inverse Laplace transform (T2-ILT).

### *T*_1_ *T*_2_ Maps

The *T*_1_ *T*_2_ correlation maps were obtained by applying the IR-CPMG (Inversion Recovery Carr–Purcell–Meiboom–Gill) pulse sequence to the samples. The number of echoes was set to 200, while the echo time was equal to 15 ms with 10 points for inversion recovery. The collected IR-CPMG data were converted to *T*_1_ *T*_2_ maps using a 2D inverse Laplace transform (2D-ILT) using in-house MATLAB codes. Ratios of *T*_1_/*T*_2_ and its reciprocal *T*_2_/*T*_1_ of the proton pool centers were written as (*T*_1_/*T*_2_)_Map_ and (*T*_2_/*T*_1_)_Map_ respectively. Ratios were manually measured from the center of the proton pools of the resulting *T*_1_ *T*_2_ maps. *T*_1_ *T*_2_ maps were analyzed using MATLAB software (ver. R2025a, The MathWorks Inc., 2025).^67^

### Circular Dichroism

Circular dichroism (CD) measurements were performed by using a Jasco J 1500 spectropolarimeter (Jasco Co., Tokyo, Japan) with a temperature control unit. Measurements were taken in the UV region between 190 and 300 nm with a resolution of 1 nm. Samples were put inside quartz cuvettes (10 mm path lengths), and the temperature was maintained at 20 ± 1 °C. Digital integration time (DIT) was 2 s. Data pitch was set at 1 nm. Scanning speed was sustained at 200 nm/min. Solutions were diluted 400× before measurements. Three measurements were averaged to obtain the final plot.

### Statistical Analysis

All measurements were performed in triplicate and were presented as the mean value ± standard deviation (SD), unless mentioned otherwise. Statistical differences were determined by one-way analysis of variance (ANOVA) followed by Tukey’s multiple comparison post-test. Min–Max normalization was applied to make a comparison between different methods of different parameters available. Results of different methods were presented and compared to each other using the *p*-value, mean absolute error (MAE), and root-mean-square error (RMSE). Differences were considered statistically significant at *p* < 0.05. Statistical analysis was carried out with JASP software (ver. 0.19.3, Amsterdam University, Netherlands).^68^

## Results and Discussion

In order to evaluate the potential of TD-NMR in detecting and quantifying glycation, both glycated and nonglycated control samples were prepared. Glycation was induced by applying heat to a mixture of bovine serum albumin (BSA) and glucose. Three temperature–time combinations were applied: 55 °C for up to 6 days, 70 °C for up to 48 h, and 85 °C for up to 6 h. During these heating periods, samples were monitored at regular intervals to assess glycation progression, and the specified durations represent the maximum heating times. Although alternative methods such as high pressure, irradiation, ultrasonication, or electrospinning can also induce glycation,^34^ these methods are not relevant to real storage conditions, but elevated temperatures are a common and significant risk during transportation and deposition of pharmaceuticals.^69^ In preliminary trials, longer heating durations, such as 10 days at 55 °C, 72 h at 70 °C, and 12 h at 85 °C, led to solvent gelation, rendering the solutions unsuitable for further analytical measurements. Therefore, shorter heating durations were chosen in this study. BSA and glucose were selected as a model biopharmaceutical formulation as they have been used in many other studies and are easier to compare and relate to different research within the existing literature.^58–60^

In order to study glycated samples using TD-NMR, it was first necessary to confirm the presence and extent of glycation in the heated samples. Glycation occurs through the reaction of free amino groups in proteins with the carbonyl groups of saccharides. The remaining unmodified free amino groups can react with the OPA reagent in the presence of a thiol, forming 1-alkylthio-2-alkylisoindoles, which exhibits an absorbance peak at 340 nm.^70,71^ Therefore, the OPA method, as a widely used technique for the glycation analysis, was employed in this study to confirm the glycation reaction on heated samples. The results were expressed as percentages (%): water (used as the blank) was assigned a value of 0%, while nonheated control samples were set at 100%. Figure 2a illustrates the consumption of residue amino groups in heated samples at different temperatures during different time frames.

**Figure 2:**
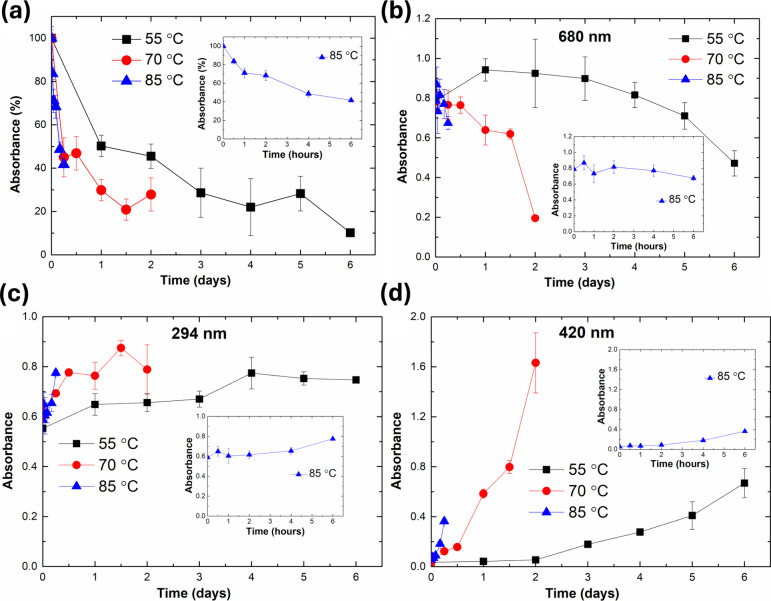
Glycation samples were heated at different temperatures (55 °C, 70 °C, and 85 °C) over time and were measured as follows: (a) Detection of free amino groups using the OPA method. (b) Protein quantification at 680 nm by the Lowry method. Absorbance of glycation products: (c) Amadori at 294 nm and (d) Melanoid at 420 nm.

Samples heated at 55 °C showed a reduction in free amino groups to approximately 15% over 6 days. Heating at 70 °C led to a 30% decrease, while exposure to 85 °C resulted in a 40% reduction within just 6 h. Increase glycation with heating for BSA and glucose has been reported in previous studies, and similar findings have also been observed with other proteins and saccharides. This outcome is expected as elevated temperatures enhance glycation reaction by promoting protein unfolding that exposing further amino groups to the reaction medium.^72,73^

### Browning Measurements

One of the simplest ways to evaluate and monitor the glycation reaction is by measuring two of its key byproducts using UV–visible spectrophotometry. The first is the Amadori product, a compound formed during the primary stages of glycation that is not visible to the naked eye but can be detected at 294 nm. The second is the Melanoid product, a brown compound formed during the later stages of glycation, which absorbs at 420 nm. Measuring both compounds provides a general indication of the progression of glycation.^74^ Figure 2c,d shows the absorbance at 294 nm (Amadori) and 420 nm (Melanoid) in samples heated at 55 °C, 70 °C, and 85 °C over time.

The levels of both Amadori and Melanoid products increased with higher temperatures and longer exposure times, consistent with the results obtained via the OPA method. The Melanoid product increased more steadily and sharply compared to the Amadori product. The increase in the Amadori product between different time points was relatively small and became significant only in the later stages. Thus, this difference between these two products unveils that the impact of high temperatures on biopharmaceuticals extends beyond reduced efficacy as it indicates that most glycation products are in advanced stages, which along with Melanoid include harmful compounds such as advanced glycation end-products (AGEs).^75,76^ This highlights the importance of detecting glycation in drugs, even in small quantities, to prevent AGEs from reaching patients. Although Browning measurements offer useful insights into the glycation process, they are somewhat ambiguous. These measurements focus on two glycation byproducts rather than providing a direct quantification of glycation levels, and the results can be influenced by factors such as particle size and protein aggregation.^34^ It is essential to keep in mind that while Browning measurement is nondestructive, it is still an invasive method that may adversely affect the drug’s efficacy; the drug container must be opened, exposing the sample to oxidation and potential contamination.

### Soluble Protein Measurements

Lowry’s method is one of the most widely recognized and employed protein quantification techniques, and it is based on the reduction of the Folin–Ciocalteu reagent and the oxidation of specific amino acids, cysteine, tryptophan, and tyrosine. This reaction results in a color change that can be quantified by measuring absorbance at 680 nm.^77,78^ Figure 2b illustrates the protein quantification of samples at 680 nm by the Lowry method.

A general decrease in the measured soluble proteins was observed following glycation. The relationship is seemingly proportional to glycation levels, as determined by the OPA method and Browning measurements. However, the decrease in protein solubility was less pronounced than the decrease observed with the OPA method. While a significant difference was observed between the initial and farthest time points of heating, changes between adjacent time intervals were minor. The reduction in soluble protein concentrations suggests protein denaturation and aggregation during glycation, which may lead to a reduction in the efficacy of biopharmaceuticals.^51^ Previous studies have demonstrated that protein solubility may either decrease or increase depending on the glycation stage but asserted that later stages of glycation lead to the formation of more insoluble glycated products.^51,66,79^ This aligns with our findings, where significant changes in solubility were detected only at specific time points. Some studies have used Lowry’s method to determine the glycation level, but it was only when protein solubility was directly affected by glycation, which is not observed in the present study.^80,81^

### TD-NMR Analysis

The fundamental premise of TD-NMR is based on the detection of NMR signals generated by the interaction of radio frequency (RF) pulses with protons. When hydrogen nuclei that are abundant in water are placed in a magnetic field, they align with the field and can be excited by an RF pulse. Once the RF pulse is turned off, the excited proton spins return to equilibrium through two distinct relaxation processes. The first is longitudinal relaxation (*T*_1_), which describes the recovery of spin energy along the direction of the magnetic field. The second is transverse relaxation (*T*_2_), which refers to the loss of coherence among proton spins in the plane perpendicular to the magnetic field.^48,82^ Figure 3a shows *T*_1_ relaxation times of samples heated at different temperatures (55 °C, 70 °C, and 85 °C) over time.

**Figure 3:**
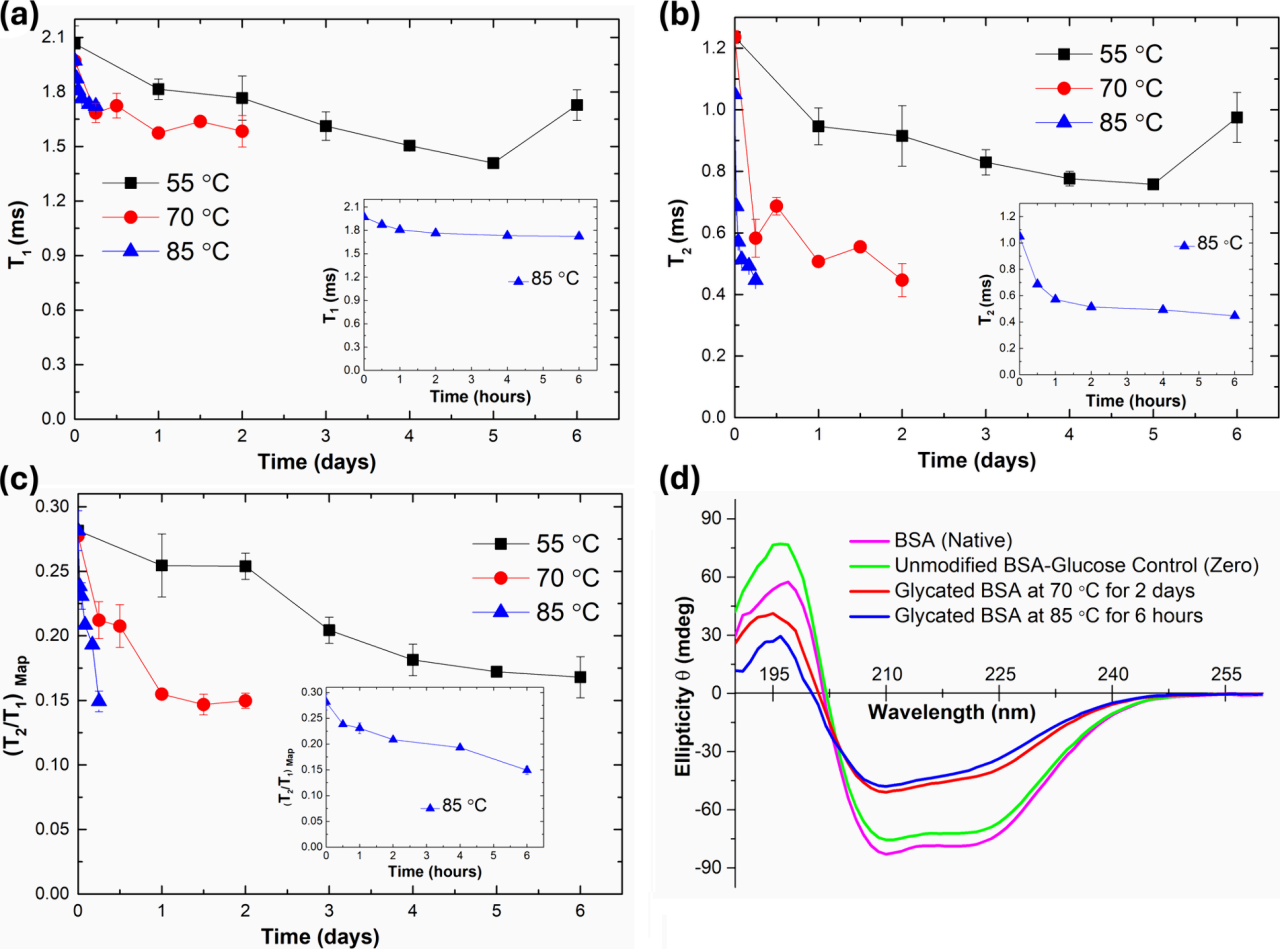
Glycation samples were heated at different temperatures (55 °C, 70 °C, and 85 °C) over time. Measurements of (a) *T*_1_ relaxation times, (b) *T*_2_ relaxation times, and (c) *T*_2_/*T*_1_ mapping values. (d) Far-UV circular dichroism spectra of native, unmodified, and glycated BSA samples at different heating conditions.

*T*_1_ relaxation times showed a decreasing trend at all temperatures. Glycation at 85 and 55 °C showed a significant and direct decrease throughout the time frame, except for the sixth day at 55 °C. The increase could be caused by sudden protein misfolding that changes the overall hydrophobicity of surface protein, or the aggregation may cause some of the byproducts to precipitate and redissolve making it easier to obtain fluctuating results. Thus, it reflects a transitional state rather than an experimental artifact. Glycation at 70 °C showed a clear decline between the control sample and the rest of the glycated samples. However, differences among the glycated samples themselves were less significant, which was similar to the results obtained from the OPA analysis. The reduction in *T*_1_ values denotes that glycation leads to decreased hydration, as glycation reacts between free amino groups and reducing saccharides, thereby removing the hydrogen bonds related to both molecules.^83,84^ These changes in hydration suggest alterations in protein conformation and stability, which in turn further highlights the negative effect of glycation on biopharmaceutics.^85,86^ Furthermore, the various pathways of the glycation result in different glycation byproducts, thereby producing molecules with varying hydration levels and varying free/bound water ratios, which may explain the minor fluctuations observed in *T*_1_ values.^87^ Figure 4a displays a representative for applying inverse Laplace transform to *T*_1_ for a sample heated at 70 °C for 24 h. T1-ILT showed mainly two major peaks and one minor peak. Although T1-ILT studies are limited, which makes it hard to assign peaks to other studies, one of these two major peaks was available only with samples containing saccharides, regardless of protein addition or heating. Consequently, the two major peaks suggested to be related to two different proton populations, where the first peak (1.5–2.0 s) is related to hydrogen interactions between solute surfaces and water and constitutes the majority of the spectrum. The other peak (0.4–0.9 s) is suggested to be related to hydrogen bonds in between solutes such as between saccharides or between saccharides and other solutes. Meanwhile, a smaller peak (below 0.3 s) was not available at all samples and is small enough that it is hard to differentiate with noise. Figure 3b shows *T*_2_ relaxation times of samples heated at different temperatures (55 °C, 70 °C, and 85 °C) over time.

**Figure 4:**
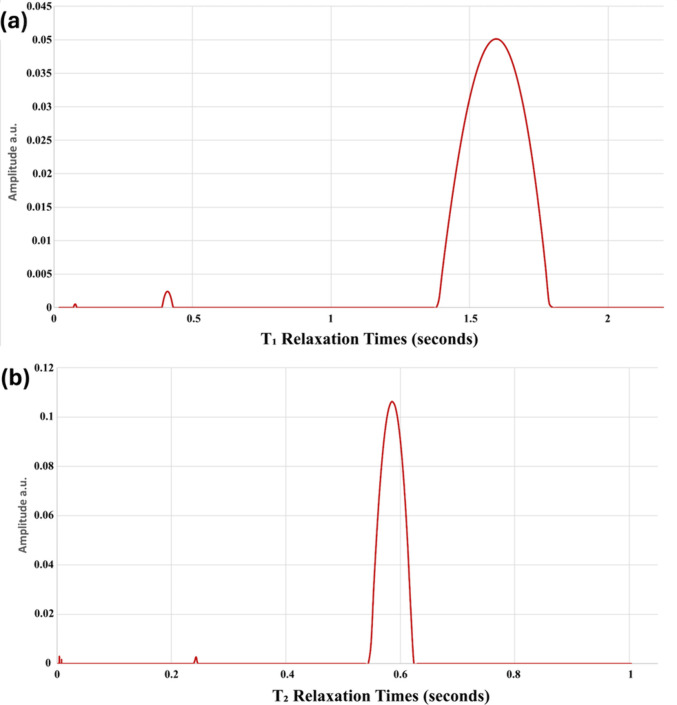
Representative for applying inverse Laplace transform to (a) *T*_1_ and (b) *T*_2_ for a sample heated at 70 °C for 24 h.

*T*_2_ relaxation times decreased with an increasing temperature and exposure duration. This decline reflects reduced mobility and freedom of water molecules and reflects a decline in the interaction between water and the surrounding molecules.^88–90^ The rate of change in *T*_2_ values varied across different time points, which may be attributed to the diverse effects of glycation on the molecular structure. For example, glycation can induce agglomeration and fibril formations that may result in decreased water mobility. Another possible explanation for the decline in *T*_2_ relaxation time is the unveiling of hydrophobic amino acids by structural changes caused by glycation. These hydrophobic compounds have less interaction with water and would reduce the *T*_2_ relaxation times.^91,92^ One important aspect of reducing the water mobility is that it may enhance drug stability and prevent crystallization, suggesting that glycation may have beneficial effects under certain conditions.^93^ However, results from previous studies using glycated soy protein isolates in food applications have been inconsistent. While using freeze-drying and spray-drying methods caused a decrease in *T*_2_ relaxation times, using a microwave-based glycation method led to an increase.^62,66^ Further research should be conducted to understand whether the glycation method itself or the drying process is the primary factor influencing the hydration behavior. T2-ILT showed one major peak in between 0.4 and 1.2 s and was aligned with *T*_2_ relaxation times measured earlier. This peak constitutes the majority of the spectrum and represents free water and water–solute interactions.^44,89,94^ Smaller peaks below 0.3 s varied in placement and intensities between different samples, and their effect is not significant for the T_2_ relaxation times. These peaks represent water restricted because of entrapment of aggregation or restricted by interacting with hydrophobic regions of solutes.^44,89^ Their variability is likely related to complexity of the glycation pathway,^95^ in addition their small sizes make them more susceptible to noise, limiting quantitative interpretation. Figure 4b displays a representative for applying an inverse Laplace transform to *T*_2_ for a sample heated at 70 °C for 24 h.

The *T*_1_ *T*_2_ map is a two-dimensional NMR technique (2D-NMR) that provides detailed insights into water domains and proton distributions by correlating *T*_2_ relaxation times (*x*-axis) with *T*_1_ relaxation times (*y*-axis). This approach is quite effective for distinguishing the contributions of several domains that contain hydrogen molecules with different proton populations. It reduces peak overlap and provides more correct information than 1D-NMR.^96^ Figure 5a shows a representative *T*_1_ *T*_2_ map of a glycated sample that displays two prominent proton pools, α and β.

**Figure 5:**
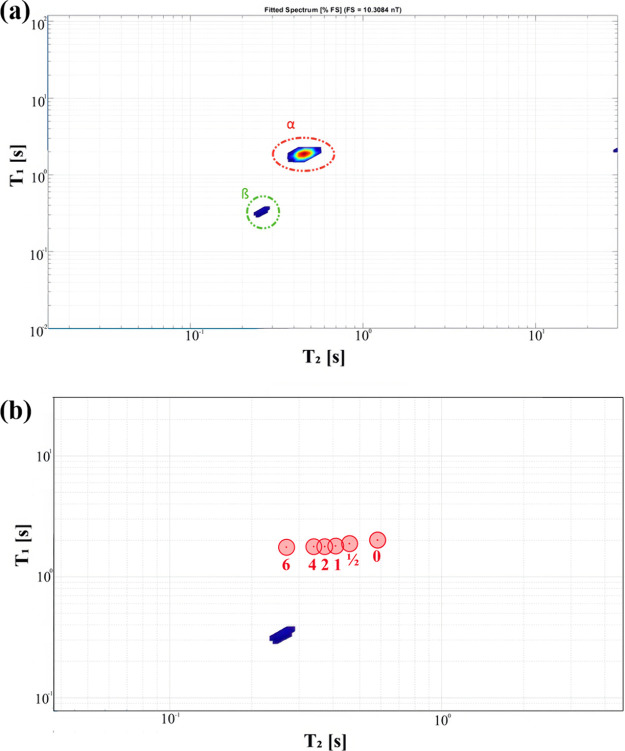
(a) Representative *T*_1_ *T*_2_ map for the sample glycated for 30 min at 85 °C. (b) Representative for the center of α proton pool of heated samples at 85 °C throughout 0–6 h.

The α pool is associated with complex polymer–water interactions, and a clear shift of its placements through glycation was observed (Figure 5b). This shift toward a lower *T*_1_/*T*_2_ ratio is attributed to the effects of glycation on TD-NMR, as glycation involves a series of reactions that reduce the overall hydrogen bonding on solutes surfaces, alongside glycation causes an increase in solute hydrophobicity and aggregation.^45,51,56^ These in turn reduce hydration and water mobility and cause reduction on *T*_1_ and *T*_2_ values. Meanwhile, the β pool appeared only in the presence of saccharides, and its placement and intensity were not persistent even between replicants. It is believed that this proton pool reflects hydrogen bonding between saccharides and each other and between saccharides and glycation byproducts. It is more likely β proton pools are in continuous change because of enantiomeric reformation of glucose and because of changes in glycation’s byproducts. Thus, only placements of the α pool were measured in this study. Ratios of *T*_1_/*T*_2_ and its reciprocal *T*_2_/*T*_1_ of the proton pool centers were written as (*T*_1_/*T*_2_)_Map_ and (*T*_2_/*T*_1_)_Map_, respectively.

The (*T*_1_/*T*_2_)_Map_ and (*T*_2_/*T*_1_)_Map_ value of a proton pool can help identifying its molecular environment, even if its placement or its *T*_1_ and *T*_2_ values have changed.^97^ This means that a change in the ratio corresponds to a shift in the pool’s location on the map, which in turn reflects an alteration in the underlying chemical structure. *T*_1_ *T*_2_ maps were employed to identify oxidation products,^82^ to study the different polymer components in water,^98^ and to study the ratio between two different drugs in a mixture.^99^ In all of these examples, ratios of *T*_1_ *T*_2_ maps were used as an identification method, whereas in the present study, they are exploited as a quantitative measurement approach. In the present study, (*T*_2_/*T*_1_)_Map_ values were measured and are shown in Figure 3c to monitor the chemical transitions associated with glycation.

Measuring the *T*_2_/*T*_1_ ratio of the α proton pool revealed a gradual decrease over time, indicating alterations in the pool’s characteristics and corresponding changes in the underlying chemical structure. These findings are consistent with other analytical results, including OPA analysis, Browning measurements, and individual *T*_1_ and *T*_2_ relaxation data.

### Structural Analysis

Circular dichroism (CD) spectroscopy is a widely used technique for identifying and analyzing the chirality of proteins, and CD provides insights into the secondary and tertiary structures of proteins.^100,101^ Far-UV CD analysis reveals information about the protein’s secondary structure, including the proportions of α-helix, β-sheet, and random coil conformations. CD spectroscopy was employed in this study to investigate changes in the secondary protein structures in BSA during glycation. Figure 3d shows far-UV CD spectra of native, unmodified, and glycated BSA samples at different heating conditions.

All samples displayed two characteristic negative peaks at approximately 209 and 222 nm. These peaks correspond to the π → π* and n → π* transitions of the peptide bond, respectively, and are typical indicators of a α-helix. This observation aligns with previous studies on both unmodified and glycated BSA, confirming that BSA predominantly consists of the α-helix.^102,103^ Glycated BSA samples demonstrated smaller peaks at these wavelengths, which indicates a decrease in the α-helix content structure and an increase in the β-sheet content and partial unfolding of the protein. This change is likely caused by the glycation-induced disruption of intramolecular hydrogen bonds within α-helices and the formation of new intermolecular hydrogen bonds that stabilize β-sheet structures. Similar trends have been observed in other glycation studies.^104–105^ These CD results further support the relationship among glycation, hydrogen bonding, and findings from TD-NMR measurements. Additionally, this further supports the relationship between glycation, hydrogen bonding, and findings from TD-NMR measurements. Additionally, the difference observed between native BSA and the unmodified BSA–glucose control (zero) may be attributed to the stabilizing effect of glucose.

### Statistical Analysis of TD-NMR

The main objective of this study was to evaluate the applicability of TD-NMR as an alternative method for detecting and quantifying glycation in pharmaceutical formulations. To assess the performance of TD-NMR techniques, their results were compared with those of the OPA method, a widely accepted reference assay for glycation. Additional comparisons were made using Browning measurements, and the soluble protein content to determine whether TD-NMR provides advantages over these traditional methods. Statistical evaluation was performed using standard regression metrics, including mean absolute error (MAE) and root-mean-square error (RMSE).^106,107^ Table 1 presents the performance metrics of each method relative to the OPA method.

**Table 1:** Correlation between Measurements of the OPA Method toward TD-NMR, Browning, and Lowry Measurements

measurements	heat temp.	*p-*value	MAE	RMSE
Browning (420 nm)	55 °C	0.15	0.51	0.62
	70 °C	0.38	0.44	0.61
	85 °C	<0.01	0.56	0.69
1/Browning (420 nm)	55 °C	0.02	0.11	0.14
	70 °C	0.07	0.07	0.07
	85 °C	<0.01	0.08	0.11
Browning (294 nm)	55 °C	0.06	0.57	0.65
	70 °C	0.05	0.48	0.66
	85 °C	0.36	0.49	0.64
1/Browning (294 nm)	55 °C	<0.01	0.18	0.22
	70 °C	<0.01	0.15	0.18
	85 °C	<0.01	0.26	0.29
*T*_1_	55 °C	<0.01	0.13	0.20
	70 °C	<0.01	0.08	0.08
	85 °C	<0.01	0.13	0.15
*T*_2_	55 °C	<0.01	0.17	0.21
	70 °C	<0.01	0.08	0.09
	85 °C	0.09	0.20	0.23
(*T*_1_/*T*_2_)_Map_	55 °C	<0.01	0.63	0.69
	70 °C	<0.01	0.57	0.75
	85 °C	<0.01	0.46	0.65
(*T*_2_/*T*_1_)_Map_	55 °C	0.02	0.1	0.13
	70 °C	0.02	0.07	0.08
	85 °C	<0.01	0.07	0.10
Lowry	55 °C	<0.01	0.40	0.45
	70 °C	0.08	0.54	0.55
	85 °C	0.15	0.23	0.29
1/Lowry	55 °C	<0.01	0.46	0.56
	70 °C	0.03	0.35	0.58
	85 °C	<0.01	0.49	0.59

Since (*T*_1_/*T*_2_)_Map_ values showed a nonproportional relationship with OPA results, their reciprocal values (*T*_2_/*T*_1_)_Map_ were also calculated to improve comparability. Similarly, Lowry and Browning measurement data were analyzed using their reciprocals for consistency since they increased; meanwhile, OPA decreased with glycation. They exhibited low correlations with the OPA results, unlike their reciprocal, which presented a statistically significant correlation with the OPA results, with *p-*values consistently below 0.05, as expected. TD-NMR measurements demonstrated a statistically significant correlation with the OPA results, with *p*-values consistently below 0.05,^108,109^ except for the *T*_2_ measurement at 85 °C, which was below 0.1. Among all tested methods, (*T*_2_/*T*_1_)_Map_ showed the highest correlation, even more than Browning measurements and other TD-NMR parameters. RMSE refers to deviations between prediction values and conventional standard values as it measures the standard deviations of errors. On the other hand, MAE represents the errors that determine variations between paired measurements that represent the same phenomena. It measures absolute differences between these measurements and how they are close to each other.^110,111^ The low RMSE and MAE values observed for (*T*_2_/*T*_1_)_Map_ confirm its low prediction error and high agreement with the OPA results. Consequently, these statistical outcomes demonstrate that (*T*_2_/*T*_1_)_Map_ exhibits a strong correlation with the OPA method, comparable to Browning analysis. Moreover, some drugs contain two or more dominant proton pools^99,112^ in which measuring changes in one pool would be more specific and accurate than measuring of *T*_1_ or *T*_2_ values. Therefore, (*T*_2_/*T*_1_)_Map_ emerges as a reliable, noninvasive, and effective technique for quantifying glycation in biopharmaceutical products.

### Limitations and Future Directions

This study demonstrates the potential of TD-NMR, particularly the (*T*_2_/*T*_1_)_Map_ measurement, as a noninvasive, low-cost, and easy-to-use method for detecting and quantifying glycation in biopharmaceuticals. By having these advantages, TD-NMR can be implemented in manufacturing environments and regulatory settings to monitor glycation and prevent glycated products from reaching patients. The results also confirm that glycation leads to reduced hydration and water mobility, reflecting structural changes in the protein conformation. However, this study has several limitations. First, the experiments were conducted under accelerated conditions, which may not represent the lower range of high temperatures in real storage environments. Future studies should include lower warm temperature conditions, such as 40 and 25 °C, and extend the exposure time with more frequent sampling points. Second, the study was conducted using model systems composed of glucose and BSA rather than commercial drug products. Commercial drugs often include different proteins and excipients such as sucrose, which would break down to glucose and cause glycation under elevated temperatures.^13^ Therefore, further investigations using commercial biopharmaceuticals are suggested.

Comparing TD-NMR to conventional methods, TD-NMR does not allow identification of specific amino acids modified by glycation unlike techniques such as LC–MS.^113^ Additionally, it is worth noting that invasive techniques, such as LC–MS, the OPA method, and boronate affinity chromatography, can directly detect and measure glycation, whereas TD-NMR can only be used as a secondary detection method. While these are beyond the scope of the current study, it highlights that TD-NMR should be considered a complementary method for glycation detection. Nevertheless, its noninvasive nature allows for the full-sample analysis without material loss or the need for additional reagents, offering clear advantages in routine quality control. It is also important to note that glycation plays a significant role not only in pharmaceutical science but also in other fields such as food science and tissue engineering.^114,115^ Future studies could explore the applicability of (*T*_1_/*T*_2_) maps for glycation analysis in these disciplines. Moreover, future studies should consider the broader application of *T*_1_ *T*_2_ mapping for detecting and quantifying chemical reactions associated with changes in hydration and molecular mobility, such as acrylamide formation, polymerization reactions, and fermentation processes.^116–118^

## Conclusions

In this study, glycation was induced through the controlled heating of a mixture consisting of bovine serum albumin and glucose. Later, glycation was validated independently using established analytical methods. The OPA analysis confirmed the progression of glycation through loss of primary amines, while Browning measurements indicated advancement toward later stages of glycation. Lowry’s method revealed aggregation, and the circular dichroism analysis demonstrated protein unfolding and a decrease in hydrogen bonding throughout glycation. One-dimensional longitudinal (*T*_1_) and transverse (*T*_2_) relaxation measurements showed that glycation leads to reduced hydration and decreased water mobility of solutes. The inverse Laplace analysis of *T*_1_ relaxation times resolved two principal proton populations: an α proton pool associated with solute–water interactions and a β proton pool related to hydrogen bonding between solutes. Two-dimensional *T*_1_ *T*_2_ mapping was subsequently applied to achieve an improved discrimination of hydration and mobility changes during glycation. In particular, *T*_2_/*T*_1_ ratios of the α proton pool were employed in order to quantify changes in the solute hydration and water mobility while minimizing contributions from dynamic saccharide rearrangements. These ratios exhibited statistically significant correlations with OPA results (*p* < 0.05, RMSE ≤ 0.12, MAE ≤ 0.10), supporting the hypothesis that TD-NMR relaxation parameters can serve as quantitative indicators of glycation progression. Overall, this work demonstrates that TD-NMR provides a noninvasive analytical method for monitoring glycation. The results further suggest that *T*_1_ *T*_2_ mapping can be extended beyond qualitative identification toward quantitative analysis of chemical processes driven by changes in the hydration and molecular mobility.

## Data Availability

Data will be made available on request.
